# Enhanced Antiplatelet Activity of Nitrated Fatty Acid Extracts from *Phaseolus vulgaris* L.

**DOI:** 10.3390/molecules31030488

**Published:** 2026-01-30

**Authors:** Lyanne Rodríguez, Héctor Leonardo Montecino-Garrido, Felipe Lagos, Basilio Carrasco, Iván Palomo, Paulina Ormazabal, Andrés Trostchansky, Eduardo Fuentes

**Affiliations:** 1 Thrombosis and Healthy Aging Research Center, VITALIS Longevity Center, Department of Clinical Biochemistry and Immunohematology, Faculty of Health Sciences, Medical Technology School, Universidad de Talca, Talca 3460000, Chile; lyrodriguez@utalca.cl (L.R.);; 2 Centro de Estudios en Alimentos Procesados-CEAP, Conicyt, Programa Regional R20F0001, Gore Maule, Talca 3460000, Chile; hector.montecino@utalca.cl (H.L.M.-G.);; 3 Escuela de Obstetricia, Facultad de Ciencias para el Cuidado de la Salud, Universidad San Sebastián, Lota 2465, Providencia, Santiago 8420524, Chile; paulina.ormazabal@uss.cl; 4 Departamento de Bioquímica and Centro de Investigaciones Biomédicas (CEINBIO), Facultad de Medicina, Universidad de la República, Montevideo 11200, Uruguay

**Keywords:** cardiovascular diseases, platelets, bean nitrated extracts, nitrated fatty acids

## Abstract

Dietary bioactive compounds are increasingly explored as complementary cardioprotective strategies, and the nitration of unsaturated fatty acids has emerged as a process capable of enhancing antiplatelet properties. This study investigated whether *Phaseolus vulgaris* L. extracts can generate nitrated fatty acids under gastric-like conditions and evaluated their effects on human platelet function. Bean extracts and major fatty acids were nitrated *in vitro* and tested using washed platelets to assess cytotoxicity, TRAP-6 and collagen-induced aggregation, activation markers (P-selectin, CD63), and mitochondrial responses including membrane potential, ROS production, and Ca^2+^ dynamics. Nitrated extracts markedly inhibited TRAP-6 induced aggregation (IC_50_ ≈ 1.8 mg/mL), whereas non-nitrated extracts showed minimal activity; this effect was reversed by β-mercaptoethanol, indicating dependence on electrophilic nitroalkenes. Fractionation revealed that the lipidic fraction accounted for most of the antiplatelet effect, and isolated nitrated fatty acids (NO_2_-LN, NO_2_-LA, NO_2_-OA) displayed stronger inhibition than their native counterparts without increasing cytotoxicity. Nitrated species additionally reduced mitochondrial membrane potential and granule secretion without elevating ROS. These findings identify *Phaseolus vulgaris* L. as a natural source of bioactive nitrated fatty acids and support their potential as nutraceutical agents capable of modulating platelet activation and contributing to cardiovascular risk reduction.

## 1. Introduction

Cardiovascular diseases (CVDs) continue to pose a significant global public health burden because of their high morbidity and mortality rates. These conditions, which include hypertension, coronary artery disease, cerebrovascular disease, heart failure, and other cardiac disorders, are among the leading causes of death worldwide [1]. The global prevalence of coronary artery disease ranges from approximately 5–8%, while peripheral artery disease affects 10–20% of the population, depending on variables such as study design, population age, sex, and geographical location [2]. The socioeconomic impact of these diseases is profound, highlighting the need for changes in clinical management through increased adherence to treatment guidelines, lifestyle modification, and pharmacological or non-pharmacological interventions [3].

Platelets play a central role in the pathogenesis of cardiovascular events. These anucleate blood components, derived from bone marrow megakaryocytes, are essential for hemostasis because of their capacity to adhere to damaged endothelium and aggregate at injury sites [4,5]. While platelet activation and aggregation are crucial for vascular repair following atherosclerotic plaque rupture, excessive or uncontrolled platelet activation can lead to thrombus formation, vascular occlusion, and subsequent ischemic events such as myocardial infarction and stroke [6]. Given the key role of platelets in thrombotic complications, targeting platelet function remains a cornerstone of pharmacological intervention [7]. In parallel, there is increasing interest in dietary strategies that can complement traditional therapies. The development of functional foods and nutraceuticals with cardioprotective properties is gaining momentum, particularly in response to the growing global prevalence of CVDs [8].

*Phaseolus vulgaris* L., commonly known as the common bean, is a staple legume recognized for its nutritional richness and health benefits. It is a major source of plant based protein (16–33%) and is especially valuable in vegetarian diets and low-income populations [9]. Besides its protein content, beans are abundant in essential vitamins (e.g., B-group vitamins, vitamin E, and vitamin C), minerals, complex carbohydrates, and bioactive compounds such as polyphenols, which contribute to antioxidant activity [10,11]. Previous studies have suggested that the cardioprotective effects of bean extracts may be linked to their antioxidant content, particularly polyphenols that can neutralize reactive oxygen species (ROS) and mitigate platelet hyperactivity [12,13]. Recent interest has emerged in enhancing the bioactivity of such extracts through processing techniques such as nitration, which may increase endogenous nitric oxide (NO) production. NO is a key regulator of vascular tone and platelet function because of its vasodilatory and antiplatelet effects [14]. Although beans are naturally low in fat, they contain essential fatty acids, notably the polyunsaturated fatty acids linoleic acid (LA) and alpha-linolenic acid (ALA), which are beneficial for cardiovascular and neurological health [15].

During digestion, unsaturated fatty acids can undergo endogenous nitration through interactions with reactive nitrogen species derived from NO oxidation. Nitrogen dioxide (NO_2_), formed from dietary nitrites in the acidic environment of the stomach, acts as a key nitrating agent in this process [16]. This reaction results in the formation of nitro-fatty acids (NO_2_-FAs), including nitro-oleic acid (NO_2_-OA), nitro-linoleic acid (NO_2_-LA), and nitro-linolenic acid (NO_2_-LN), which have been shown to modulate vascular tone, inflammation, and platelet activation [17]. Extensive experimental evidence has shown that NO_2_-FAs offer protection against various cardiovascular and metabolic insults, including atherosclerosis, cardiac ischemia–reperfusion injury, diabetes, hypertension, and vascular inflammation [18,19,20]. These compounds can attenuate endothelial dysfunction by inhibiting membrane adhesion molecule expression and reducing platelet activation [21]. Moreover, NO_2_-FAs exert cytoprotective effects under conditions of mitochondrial dysfunction by forming electrophilic adducts with critical residues in key regulatory proteins [22,23]. Their impact on mitochondrial function ranges from respiratory inhibition to protective effects in models of ischemia–reperfusion injury. Moreover, NO_2_-FAs derived from dietary sources such as extra virgin olive oil have been linked to improved mitochondrial function in non-alcoholic fatty liver disease [23].

The global rise in chronic diseases associated with sedentary lifestyles and the shift from traditional diets to those rich in refined carbohydrates and unhealthy fats underscore the need for sustainable dietary strategies to promote cardiovascular health. In this context, *Phaseolus vulgaris* L., a widely consumed, nutritionally dense legume, may serve as a valuable dietary source of NO_2_-FAs. The present study aimed to investigate the generation of NO_2_-FAs from *Phaseolus vulgaris* L. under simulated gastric conditions and to evaluate their antiplatelet properties. By exploring the potential of bean derived NO_2_-FAs, this research seeks to enhance the nutritional and therapeutic value of a widely accessible food crop in Chile and beyond.

## 2. Results

### 2.1. Effect of the Nitration Process on the Extract’s Antiplatelet Activity

Lipids within platelets do not directly trigger aggregation; instead, they play a crucial role in modulating the response to other aggregating agents. They act as fine tuning regulators, functioning either as inhibitors, such as omega-3 fatty acids, or as pro-thrombotic mediators, such as thromboxane A_2_ precursors. Since dietary lipids preferentially react with nitrating agents during digestion, changes in the biological activity of nutraceuticals are commonly observed.

**Figure 1:**
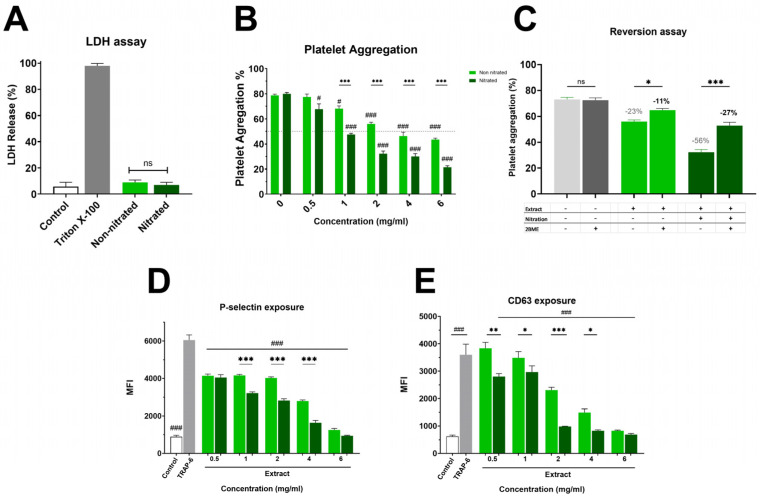
Cytotoxicity and Platelet Aggregation by Nitrated vs. Non-nitrated Extracts. (**A**) LDH release following incubation with either nitrated or non-nitrated extracts was evaluated. Green represents the non-nitrated extract, and deep green shows the nitrated extract. Triton X-100 was used as a positive control. To evaluate platelet aggregation and activation, washed platelets were pre-incubated with increasing concentrations of each extract (0.5-6.0 mg/mL) and activated with TRAP-6 (5 µM). (**B**) Platelet aggregation in response to nitrated and non-nitrated extracts at various concentrations, using the same color scheme. TRAP-6 (5 µM) served as a positive control. (**C**) Platelet aggregation was assessed after treatment with control, nitrated, or non-nitrated extracts, in the presence or absence of β-mercaptoethanol (BME). (**D**) P-selectin expression in TRAP-6 stimulated platelets following pre-incubation with nitrated and non-nitrated extracts at various concentrations. (**E**) CD63 exposure on the platelet membrane under the same conditions as in (**D**). Data are presented as mean ± SEM (n = 6 per group). The statistical significance was assessed as follows: comparison against TRAP-6 control is showed by # (# *p* < 0.05 and ### *p* < 0.001); comparison against the corresponding non-nitrated concentration is indicated by * (* *p* < 0.05, ** *p* < 0.01 and *** *p* < 0.001). “ns” shows no statistically significant difference.

Figure 1A and Supplementary Material Figure S1 show the impact on platelet viability. The non-nitrated extract induced slightly higher LDH release than the nitrated extract; however, this difference was not statistically significant, showing that neither extract caused platelet damage. Both extracts were evaluated in the platelet aggregation assay, where a marked difference in antiplatelet activity was observed (Figure 1B). The nitrated extract exhibited strong inhibition of platelet aggregation, with an IC_50_ of 1.8 ± 0.1 mg/mL. In contrast, the non-nitrated extract showed a much weaker effect, with an IC_50_ exceeding the highest concentration tested (6 mg/mL). This enhanced antiplatelet effect of the nitrated extract was also clear in granule secretion markers. As shown in Figure 1C, P-selectin externalization was lower in platelets treated with the nitrated extract compared to the non-nitrated extract. Both extracts inhibited P-selectin expression relative to the control, with the nitrated extract showing a greater inhibitory effect. Finally, Figure 1D illustrates platelet activation assessed by CD63 surface expression. The analysis suggests that the nitrated extract significantly reduces CD63 exposure, further supporting its inhibitory effect on both platelet activation and aggregation. Overall, nitration enhances the antiplatelet properties of the extract without increasing cytotoxicity in TRAP-6 activated platelets.

While the extracts significantly inhibited TRAP-6 induced aggregation, no significant effects were observed when collagen (2 µg/mL) was used as the agonist (Table S1). This finding is consistent with a preferential modulation of PAR-1 associated pathways rather than GPVI mediated platelet activation.

### 2.2. Role of Nitration Bonds on Antiplatelet Activity

The platelet aggregation data revealed a clear difference in biological activity between nitrated and non-nitrated Hallado Alemán extracts. To determine whether the presence of electrophilic nitroalkenes was responsible for the increase in antiplatelet activity, the extracts were pre-incubated with an excess of BME, followed by addition to platelets, and aggregation was evaluated (Figure 1C). Control samples with TRAP-6 plus BME showed aggregation values around 69–73%, indicating that BME alone does not influence platelet activation induced by TRAP-6, thus ruling out confounding effects on platelet function. When the nitrated extract was pre-incubated with 1 mM BME, platelet aggregation increased notably, showing a substantial loss of antiplatelet activity. This suggests that electrophilic nitroalkenes (nitrated fatty acids with the NO_2_ group at the double bond) handle the inhibition of platelet aggregation. In the presence of BME, the nitrated extract behaved similarly to the non-nitrated one. In contrast, for the non-nitrated extract, incubation with BME did not significantly alter platelet aggregation, confirming that the observed BME effect is specifically related to blocking the effects of nitrated fatty acids rather than any non-specific effects on the extract.

### 2.3. Comparative Antiplatelet Effects of Lipidic, Phenolic, and Combined Extract Fractions

**Figure 2:**
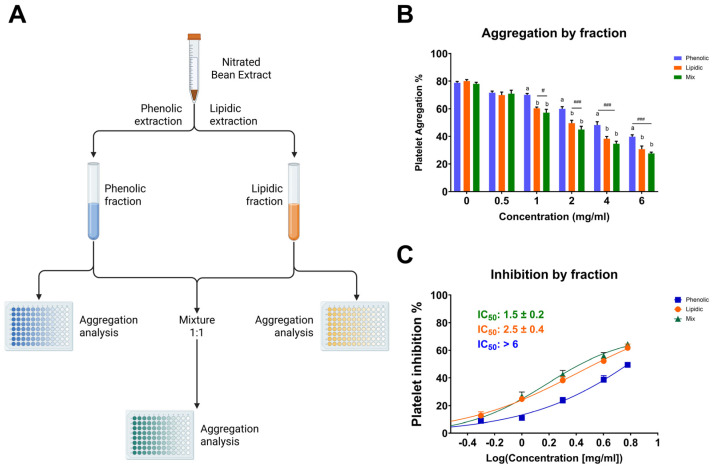
Antiplatelet activity of lipidic, phenolic, and combined (1:1) fractions of the nitrated extract. (**A**) Schematic representation of the extraction process used to obtain the phenolic, lipidic, and mixed (1:1) fractions. (**B**) Platelet aggregation induced by TRAP-6 (5 µM) in the presence of fractions at various concentrations. (**C**) IC_50_ values were calculated for phenolic, lipidic, and mixed fractions. Washed platelets were pre-incubated with increasing concentrations of each fraction (0.5-6.0 mg/mL), followed by stimulation with TRAP-6 (5 µM). Data are presented as mean ± SEM (n = 6 per group). Statistical significance was assessed as follows: comparison against TRAP-6 control is indicated by # (# *p* < 0.05, and ### *p* < 0.001). The different letters (a and b) in the column show significant differences between them according to Tukey’s test (*p* < 0.05).

Among the tested fractions, the lipidic extract exhibited the most potent antiplatelet activity, suggesting that the bioactive components responsible for inhibiting platelet aggregation are mainly concentrated in the lipidic portion (Figure 2). At a concentration of 6 mg/mL, the lipidic fraction reduced platelet aggregation to 34.2 ± 2.3%, in contrast to 43.7 ± 1.4% for the phenolic fraction and 30.7 ± 1.7% for the combination of both (lipidic and phenolic). This trend was consistent across all concentrations tested. Notably, the mixture of fractions did not show a markedly synergistic effect compared to the lipidic extract alone, reinforcing the hypothesis that the lipidic fraction contains the primary compounds responsible for the antiplatelet activity. Since this fraction was subjected to nitration, it is plausible that nitrated lipid species, such as NO_2_-FAs, play a central role in the observed bioactivity. These results underscore the significance of the nitration process in augmenting antiplatelet efficacy and reinforce the notion that targeted chemical modifications of natural lipid matrices can yield novel antithrombotic agents.

### 2.4. Determination of Fatty Acid Content of Bean Extract

As shown in Table 1, polyunsaturated fatty acids (PUFAs) account for 60.91% of the total fatty acids present in the bean extract, followed by monounsaturated fatty acids (MUFAs) and saturated fatty acids (SFAs). Figure S2 shows the chemical structure of the saturated and unsaturated fatty acids identified in the extract. The major unsaturated fatty acids in bean extract are LN, LA, and OA in that order. These results support that the bean extract is a relevant food substrate for NO_2_-FAs formation under acidic gastric conditions, impacting its nutraceutical properties.

### 2.5. Antiplatelet Activity of Major Nitrated Fatty Acids in the Bean Extract

**Figure 3:**
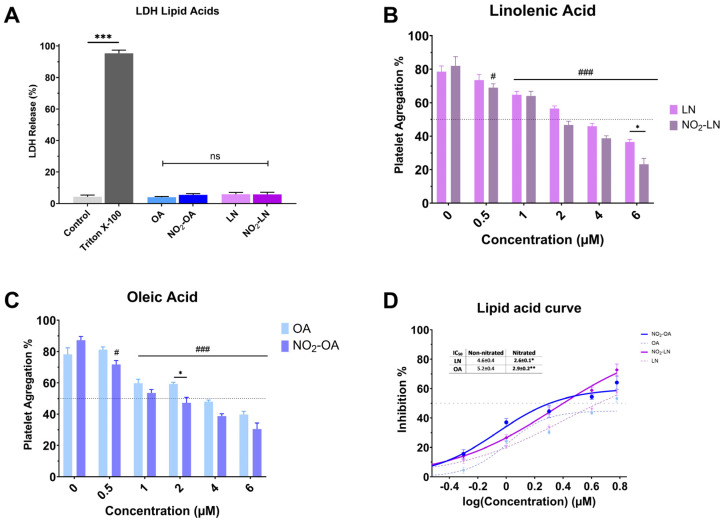
Effect of isolated nitrated fatty acids on platelet aggregation. (**A**) LDH release from platelets exposed to nitrated and non-nitrated fatty acids. (**B**,**C**) correspond to the dose-dependent evaluation of platelet aggregation of NO_2_-LN and NO_2_-OA. (**D**) IC_50_ values were calculated for major nitrated and non-nitrated fatty acids. Washed platelets were pre-incubated with increasing concentrations of each compound (0.5–6.0 mg/mL), followed by stimulation with TRAP-6 (5 µM). Data are presented as mean ± SEM (n = 6 per group). Statistical significance was assessed as follows: comparison against TRAP-6 control is indicated by # (# *p* < 0.05, and ### *p* < 0.001); comparison of the nitrated vs. non-nitrated concentration is indicated with * (* *p* < 0.05, ** *p* < 0.01 and *** *p* < 0.001). “ns” indicates no statistically significant difference.

Nitration alters the chemical characteristics of fatty acids, potentially increasing their bioactivity. Nitrated fatty acids have been shown to have a variety of biological effects, including antibacterial, anti-inflammatory, and vasodilator properties. We analyzed the cytotoxicity of individual NO_2_-FAs (Figure 3A). Nitration does not increase the toxicity levels of the NO_2_-FAs since the levels of free LDH (%) obtained are significantly lower (*p* < 0.001) when compared to the positive control (Triton X-100). The antiplatelet activity of each nitrated PUFA was higher compared to the non-nitrated fatty acid (Figure 3B–D).

### 2.6. Mitochondrial Effect of Nitrated Bean Extract

**Figure 4:**
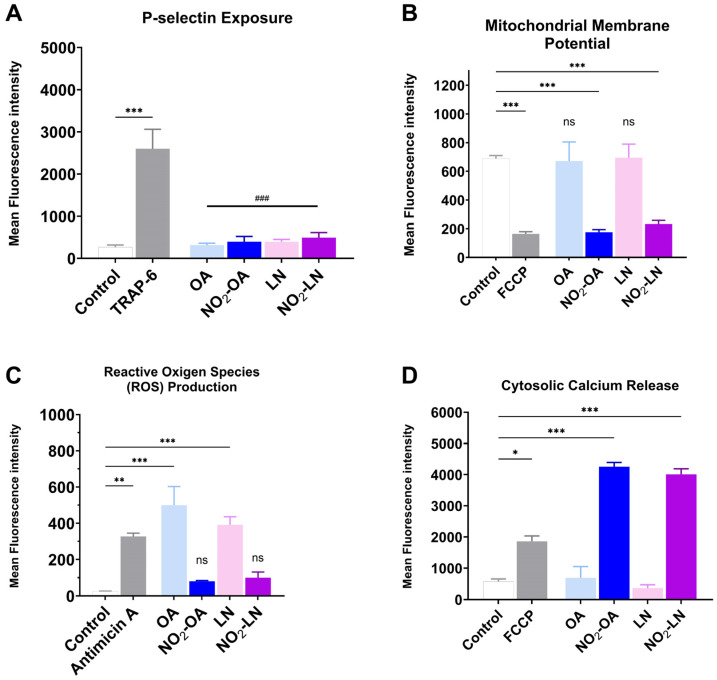
Effect on mitochondrial dysfunction of the nitrated fatty acids present in the Hallado Alemán extract. (**A**) P-selectin exposure induced by incubation with LN, OA, NO_2_-LN, and NO_2_-OA was evaluated by flow cytometry. Positive control corresponds to the condition of TRAP-6-activated platelets. (**B**) Mitochondrial membrane potential (MMP) modulation was determined as explained in the Methods section, with FCCP as a positive control. (**C**) Reactive oxygen species (ROS) production with Antimicin A as a positive control. (**D**) Calcium levels induced by fatty acids with FCCP as a positive control were also determined. Washed platelets were pre-incubated with the IC_50_ concentration of compounds (OA, NO_2_-OA, LN, and NO_2_-LN), followed by stimulation with TRAP-6 (5 µM). Data are presented as mean ± SEM (n = 6 per group). The statistical analysis was performed using one-way ANOVA followed by Tukey’s post hoc test. * *p* < 0.05; ** *p* < 0.01 and *** *p* < 0.001. ### *p* < 0.001 versus TRAP-6 stimulated platelets (comparison of nitrated and non-nitrated fatty acids with the agonist). “ns” indicates no statistically significant difference. FCCP: carbonyl cyanide p-(trifluoromethoxy)phenylhydrazone. Control corresponds to DMSO 0.2% (vehicle).

The association between cardiovascular disease (CVD) development and mitochondrial dysfunction is well established. Several NO_2_-FAs have been reported to modulate mitochondrial metabolism, either by enhancing mitochondrial efficiency or by inhibiting specific respiratory complexes. In this context, we evaluated whether the nitrated fatty acids tested in this study influence platelet activation and mitochondrial related parameters under pro-aggregatory conditions. All results shown in Figure 4A–D were obtained using TRAP-6 activated platelets following pre-incubation with the shown nitrated fatty acids, ensuring consistency across experimental readouts.

Figure 4A shows how each type of NO_2_-FAs tested reduces granule secretion, as observed by P-selectin membrane exposure. The effect on mitochondrial membrane potential, shown in Figure 4B, shows that both NO_2_-OA and NO_2_-LN decrease the mitochondrial potential, without increasing intracellular ROS (Figure 4C). Interestingly, Ca^2+^ homeostasis was affected by a significant increase (Figure 4D), which did not induce aggregation.

## 3. Discussion

Excessive platelet aggregation and thrombus formation are well-established risk factors for CVD, including coronary artery disease and stroke [24]. Recent attention has focused on NO_2_-FAs as promising antiplatelet agents with potential therapeutic implications [25,26]. In this study, we investigated the antiplatelet properties of NO_2_-FAs derived from *Phaseolus vulgaris* L. (common bean), with a focus on the subspecies Hallado Alemán, which is characterized by a favorable PUFAs profile [27].

The fatty acid composition of *Phaseolus vulgaris* L. is influenced by cultivation conditions and extraction methods. Our gas chromatography/mass spectrometry (GC/MS) analysis confirmed that the bean lipid profile is dominated by LN (33.2 g/100 g), LA (27.7 g/100 g), and OA (3.8 g/100 g), accounting for approximately 66% of total fatty acids. These findings support the potential of *Phaseolus vulgaris* L. as a dietary source of beneficial PUFAs, which confer cardioprotective effects.

Gastric nitration under acidic conditions facilitated the generation of NO_2_-FAs from these PUFAs. *In vitro* synthesis enabled controlled production of these bioactive compounds for further characterization [28]. NO_2_-FAs are known for their anti-inflammatory and antioxidant properties; here we provide evidence for their antiplatelet activity. Platelet aggregation induced by thrombin receptor-activating peptide (TRAP-6) was significantly inhibited by nitrated extracts in a concentration-dependent manner, with a stronger effect than non-nitrated controls. Interestingly, the extracts had minimal impact on collagen-induced aggregation, suggesting selective inhibition of G-protein-coupled receptor (GPCR) pathways, such as those mediated by protease-activated receptor-1 (PAR-1), rather than GPVI mediated collagen signaling.

These findings are consistent with those reported by Coles et al. [29], who showed that NO_2_-LA inhibits thrombin mediated platelet activation by increasing intracellular cAMP levels and reducing calcium mobilization. The receptor selectivity observed in our study, whereby nitrated extracts selectively inhibited TRAP-6 induced aggregation but not collagen induced responses, supports the hypothesis that nitrated compounds derived from *Phaseolus vulgaris* L. preferentially modulate thrombin receptor-associated signaling pathways, as previously described for purified nitrated fatty acids. Such selectivity may be advantageous, as it allows modulation of platelet activation without broadly suppressing collagen-dependent hemostatic mechanisms.

Mechanistically, the inhibitory effect was reversed by BME, a reducing agent that disrupts nitroalkene moieties, confirming the specificity of nitration in mediating the biological activity. The non-nitrated extract showed no such reversal, reinforcing this conclusion. Fractionation of the extract further revealed that the lipid fraction was primarily responsible for the antiplatelet activity, achieving inhibition levels of ~58% at 6 mg/mL. In contrast, the phenolic fraction showed only modest effects. Notably, co-administration of both fractions did not produce additive or synergistic inhibition, showing that phenolic compounds may not significantly enhance the bioactivity of NO_2_-FAs in this context. NO_2_-FAs also suppressed key markers of platelet activation, including P-selectin and CD63, in a concentration dependent manner. While previous studies have reported that NO_2_-FAs may act through nitric oxide-related mechanisms [30,31], the present study does not directly address NO production or signaling. Therefore, alternative or complementary mechanisms such as modulation of phospholipase A_2_ activity, integrin GPIIb/IIIa signaling, or other redox-sensitive pathways may contribute to the observed effects and warrant further investigation [32].

**Figure 5:**
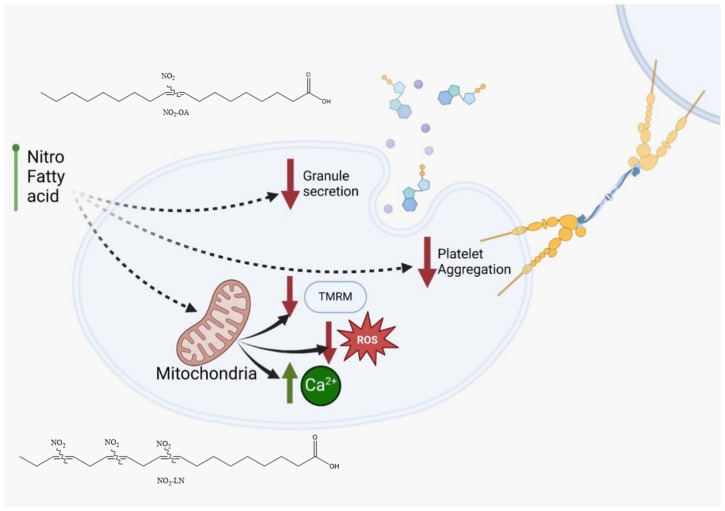
NO_2_-FAs identified in *Phaseolus vulgaris* L. are proposed to possess antithrombotic properties based on their ability to inhibit platelet activation and attenuate mitochondrial membrane potential (TMRM) elevation without inducing reactive oxygen species (ROS) release. NO_2_-OA: nitro-oleic acid; NO_2_-LN: nitro-linolenic acid.

Among the fatty acids evaluated, LN emerged as a key contributor to the antiplatelet effects observed, consistent with its abundance in Hallado Alemán beans. Our data support the hypothesis that LN, particularly when nitrated, significantly contributes to the bioactivity of the extract. This aligns with other studies linking dietary PUFAs to reduced inflammatory and thrombotic activity [33]. The interaction of NO_2_-FAs with other bioactive compounds, such as resveratrol or polyphenols found in red wine and apples, has been reported to potentiate their antiplatelet effects [34]. Similar synergy might be explored in future studies involving dietary matrices rich in antioxidants and PUFAs. Notably, we observed that NO_2_-FAs from *Phaseolus vulgaris* L. inhibited platelet activation and mitochondrial membrane potential elevation without increasing ROS production (Figure 5). This paradoxical finding suggests that NO_2_-FAs may function as mild mitochondrial uncouplers or selectively modulate components of the electron transport chain. Similar effects have been reported for NO_2_-OA, which shifts mitochondrial metabolism toward glycolysis. This mitochondrial reprogramming may underlie some of the protective effects of NO_2_-FAs against oxidative stress and thrombotic risk.

Importantly, Hallado Alemán beans offer a cost effective and widely available dietary source of PUFAs and NO_2_-FAs, potentially providing a practical alternative to more expensive sources such as olive oil or specialized supplements. However, despite promising *in vitro* data, several limitations must be addressed. Our models employed platelet-rich plasma and washed platelets, which do not fully replicate the complexity of whole blood physiology. Some assays used supra-physiological concentrations of extracts, and sample sizes were limited. Therefore, future studies should include *in vivo* validation, pharmacokinetic analyses, and dose–response evaluations under physiologically relevant conditions. Explaining the precise molecular pathways by which NO_2_-FAs exert their effects will also be critical for advancing their clinical development. These insights will be essential to establishing whether *Phaseolus vulgaris* L. derived NO_2_-FAs can serve as effective, food-based interventions for CVD prevention and therapy.

### Experimental Limitations and Challenges for Physiological Extrapolation

A rigorous interpretation of the present findings requires acknowledgment of several methodological and translational limitations that constrain the extent to which the results can be generalized. Although direct mass spectrometry based characterization of nitrated species in the whole extract was not performed, several converging lines of evidence support the occurrence of fatty acid nitration and its contribution to the enhanced antiplatelet activity observed. Specifically, the nitrated extract exhibited a markedly greater inhibitory effect on platelet aggregation than the non-nitrated control, showing that the nitration process increased biological activity. Pre-incubation of the nitrated extract with β-mercaptoethanol completely abolished its antiplatelet effect, a response consistent with the presence of electrophilic nitroalkene moieties known to undergo thiol dependent Michael addition reactions. This characteristic property of nitrated unsaturated fatty acids, together with the enrichment of activity in the lipid fraction and the ability of previously synthesized and fully characterized nitrated fatty acids (e.g., NO_2_-LN and NO_2_-OA) to reproduce the observed effects, provides strong indirect chemical and biological evidence for nitration. While direct LC–MS/MS or GC–MS analyses would strengthen structural confirmation, the combined use of a validated nitration method, thiol reactivity assays, lipid fractionation, and comparative biological analyses supports the conclusion that nitration of unsaturated fatty acids contributes to the potentiated antiplatelet activity of the extract.

There is an inherent discrepancy between the effective concentrations identified *in vitro* and those realistically achievable *in vivo*. Functional assays, including platelet aggregation, activation markers, and mitochondrial bioenergetics, required concentrations of extracts or nitroalkene species that may exceed physiologically attainable levels derived from dietary intake. This discrepancy introduces uncertainty when extrapolating parameters such as IC_50_ to clinically relevant scenarios. The IC_50_ values reported here should be interpreted as comparative, system dependent parameters reflecting inhibitory behavior under the specific experimental conditions, rather than as absolute potency constants of individual chemical species. Therefore, complementary studies examining bioavailability, metabolism, and pharmacokinetics are essential to determine maximal plasma concentrations and to assess the potential endogenous formation of NO_2_-FAs under gastrointestinal conditions [35].

A second limitation arises from the use of simplified *in vitro* models, such as washed platelets and platelet-rich plasma, which do not fully capture the complexity of the human circulatory environment. Physiological modulators, including interactions with erythrocytes and leukocytes, plasma protein binding, vascular shear stress, and hepatic biotransformation, can markedly influence platelet and mitochondrial responses. As a result, the magnitude and even the direction of the effects observed *in vitro* may differ under physiological conditions. This underscores the need for future studies employing whole blood under shear flow, *ex vivo* perfusion systems, and *in vivo* animal models to improve the ecological validity of the findings [36].

Another limitation relates to the intrinsically low lipid content of *Phaseolus vulgaris* L., which restricts the extractive yield of PUFAs and consequently limits the formation of bioactive NO_2_-FAs. Although the lipid profile dominated by ALA, LA, and OA supports the generation of nitroalkene species, the absolute amount of lipids is low and subject to substantial variability across cultivars, environmental conditions, and processing methods. Previous studies have shown that NO_2_-FA formation depends critically on both lipid abundance and the redox environment [37]. Thus, the food matrix itself may inherently restrict endogenous NO_2_-FA generation *in vivo*. Strategies such as lipid enrichment, germination, enzymatic hydrolysis, or lipid fractionation should be explored to enhance PUFA bioavailability and nitration potential [38].

Finally, the absence of deeper mechanistic analyses including redox proteomics, identification of molecular targets, and validation in animal models limits our ability to fully explain the signaling pathways modulated by NO_2_-FAs and their potential effects on inflammation, redox homeostasis, platelet activation, and endothelial function. Previous studies show that nitrated fatty acids can modulate key regulatory pathways such as NF-κB, Nrf2, and eNOS, highlighting their biological relevance; however, systematic validation under physiological conditions remains necessary [39,40]. Together, these limitations highlight the need for future multidisciplinary research to better understand the physiological relevance, therapeutic potential, and practical applications of NO_2_-FAs derived from *Phaseolus vulgaris* L.

## 4. Materials and Methods

### 4.1. Chemicals

Thrombin receptor activating peptide 6 (TRAP-6), Antimycin (AA), dihydroethidium (DHE), intracellular calcium fluorescence indicator (Fluo-4-AM), and trifluoromethoxyphenylhydrazone (FCCP) were obtained from Sigma-Aldrich (St. Louis, MO, USA). Anti-CD62P-PE, anti-CD61-FITC, and anti-CD63-PE antibodies were purchased from BD Pharmingen (BD Biosciences, San Diego, CA, USA). The HPLC-grade solvents used were purchased from Burdick and Jackson (Muskegon, MI, USA).

### 4.2. Extraction Process of the Bean Sample

To obtain the extracts, the extraction was performed using a bean to water ratio of 1:40 for 1 h. Ultrasound-assisted extraction (UAE) was conducted at 50% amplitude (10 kHz) for a sonication duration of 60 min. The resulting samples were then centrifuged at 3500 rpm for 15 min at 20 °C. The supernatant was subsequently filtered using Corning Falcon cell filters with pore sizes of 100, 70, and 40 µm, respectively. The filtered supernatant was then frozen at −86 °C for 48 h and subsequently lyophilized (Operon, FDU 7024, Gimpo, South Korea) for 20 h before analysis [12].

### 4.3. Fatty Acid Composition of Bean Extract

The fatty acid composition of *Phaseolus vulgaris* L. was determined using gas chromatography. A minimum sample quantity of 2 g was required for analysis. Lipid extraction followed the AOAC Official Method 996.06 for Fat (Total, Saturated, and Unsaturated) in Foods, with specific modifications [41]. Briefly, 2 g of the sample was weighed, and a solution containing 2 mL of acidified methanol and 1 mL of toluene was added. The samples were subjected to rotary tube shaking at 1700 rpm for 10 min and then placed in a water bath at 90 °C for 2 h, with temperature control during the initial 30 min. After this, the samples were cooled to room temperature, mixed with 5 mL of saturated NaCl, and centrifuged for 8 min at 4000 rpm. The toluene phase obtained was then injected into the gas chromatography system for further analysis.

Fatty acid composition analysis was performed by gas chromatography using a flame ionization detector (FID) maintained at 285 °C. Separation was achieved using a 100 m × 0.25 mm capillary column with a 0.20 µm bis-cyanopropyl stationary phase (HP-88 or SP-2560). The oven temperature program started at 100 °C with a 4 min hold, followed by a ramp of 3 °C/min to 240 °C, which was maintained for 15 min.

### 4.4. Nitration of the Bean Extract and Its Major Fatty Acids

Lyophilized bean extract (*Phaseolus vulgaris*) (100 mg) was transferred to a glass reaction tube and resuspended in 1.0 mL of sodium phosphate buffer adjusted to pH 3.0 to simulate acidic gastric conditions. The suspension was vortex-mixed for 1 min to ensure homogenization. A magnetic stirring bar was added, and the tube was placed in a water bath on a magnetic stirrer maintained at 37 °C. The temperature of the water bath was gradually increased and monitored until reaching 41 °C [42].

Before initiating the reaction, dissolved oxygen was removed by purging the reaction mixture, and the glass tube was immediately sealed to maintain an oxygen-depleted environment. The nitration reaction was initiated by the addition of sodium nitrite (NaNO_2_) to reach a final concentration of 5 mM, followed by continuous stirring at 37 °C. The reaction mixture was maintained under these conditions for a total reaction time of 60 min. Control samples (non-nitrated extracts) were processed in parallel under identical experimental conditions, except that NaNO_2_ was omitted. In addition, the major fatty acids oleic acid (OA), linoleic acid (LA), and linolenic acid (LN) were nitrated and extracted as previously reported [16,28].

#### Extraction of Nitrated Lipids

Following completion of the nitration reaction, nitrated lipids were extracted using an organic solvent system. Briefly, 2.5 mL of a hexane/isopropanol/acetic acid mixture was added to each reaction tube using glass pipettes, and the mixture was vortexed for 1 min. Subsequently, 2.5 mL of hexane was added, followed by vortex mixing for an additional 1 min. Samples were then centrifuged at 1800 rpm for 5–6 min to achieve phase separation.

The upper organic phase was carefully collected and transferred to clean glass tubes. The remaining aqueous phase was re-extracted with an additional 2.5 mL of hexane, vortexed for 1 min, and centrifuged under the same conditions. The organic phases were combined and evaporated to dryness under controlled conditions, and the resulting extracts were weighed to determine the extraction yield. Dried samples were stored at −20 °C until further analysis [21,43].

### 4.5. Preparation of Human Platelets

A total of six healthy volunteers aged 20–65 years were recruited for this study. Inclusion criteria consisted of individuals with no clinical history of cardiovascular, metabolic, or hematological diseases. Exclusion criteria were: (i) use of any medication, specifically antiplatelet or anti-inflammatory drugs (e.g., NSAIDs), within 7 days before blood collection; (ii) smoking; and (iii) consumption of dietary supplements that could interfere with platelet function. All participants provided written informed consent before blood withdrawal, and the study was conducted in accordance with the Declaration of Helsinki and approved by the Local Ethics Committee (Protocol 16-2023) [44]. After reading and signing the informed consent, 20 mL venous blood samples were collected by phlebotomy using a 21 G needle [5]. Each sample (5 mL) was centrifuged using a DCS-16 Centrifugal Presvac RV centrifuge (Presvac, Buenos Aires, Argentina) at 240 g for 10 min to obtain platelet-rich plasma (PRP). Two-thirds of the PRP was then removed, and the remaining portion was centrifuged at 650× *g* for 10 min to obtain platelet-poor plasma (PPP). The remaining 5 mL was processed similarly, first centrifuged to obtain PRP and then further centrifuged at 650× *g* for 10 min. The pellet was washed with HEPES-Tyrode’s buffer containing Prostaglandin E1 (PGE1, 120 nmol/L). PRP and washed platelets were adjusted to a concentration of 200–300 × 10^6^ platelets/mL using a Bayer Advia 60 Hematology System (Bayer Diagnostics, Tarrytown, NY, USA). Data are expressed as the mean ± standard error of the mean (SEM) from at least six independent experiments (n = 6), each performed using platelet samples obtained from different healthy volunteers.

### 4.6. Cytotoxicity of Extracts

Washed platelets (3 × 10^8^ platelets/mL) were incubated for 10 min at 37 °C with the highest concentration of extracts tested (6 mg/mL). Then, platelets were centrifuged at 800× *g* for 8 min, and the resulting supernatant was analyzed with the lactate dehydrogenase (LDH) cytotoxicity assay kit (Cayman Chemical, Ann Arbor, MI, USA). The absorbance of the reaction was measured at 490 nm in a microplate reader (Microplate Reader (Thermo Scientific Multiskan^TM^ Go, Thermo Fisher Scientific, Vantaa, Finland), with 10% TritonX-100 used as a positive control [45].

### 4.7. Inhibition of Platelet Aggregation of Bean Extract and Fatty Acids

Platelet aggregation was assessed using a changed microplate assay. Washed platelets were incubated with nitrated or non-nitrated *Phaseolus vulgaris* L. (bean) extracts at varying concentrations (0.5, 1, 2, 4, and 6 mg/mL) or vehicle control (0.2% DMSO) at 37 °C for 6 min. Aggregation was then stimulated with either TRAP-6 (5 μM) or collagen (2 μg/mL). Samples were transferred to 96-well plates and shaken at 1200 rpm for 5 min at 37 °C. Platelets treated with 0.2% DMSO were used as the control for maximal aggregation. All treatments were prepared using DMSO as a vehicle to ensure equivalent solvent conditions across experimental groups; the final DMSO concentration did not exceed 0.2% (*v*/*v*), a level confirmed not to affect platelet viability or function. Absorbance was measured at 450 nm using a microplate spectrophotometer (Thermo Scientific Multiskan™ GO, Thermo Fisher Scientific, Vantaa, Finland). To determine the concentration required to inhibit 50% of platelet aggregation (IC_50_), dose–response curves were constructed for both nitrated and non-nitrated bean extracts. To determine the half-maximal inhibitory concentration (IC_50_), dose–response curves were generated by fitting the experimental data to a nonlinear regression model (log[inhibitor] vs. normalized response with variable slope) using GraphPad Prism 9.0 (GraphPad Software Inc., San Diego, CA, USA). Each IC_50_ value corresponds to the concentration required to inhibit 50% of the maximal platelet aggregation response and is expressed as the mean ± SEM from six independent experiments (n = 6). A concentration range of 0.5 to 6 mg/mL was selected for the extracts based on previous studies on lipid bioactivity, allowing evaluation from minimal inhibition to saturation of the inhibitory effect [42].

Additionally, the antiplatelet activity of individual nitrated fatty acids identified in the extracts OA, LA, and LN was evaluated [42,46]. To investigate the role of nitration in the biological activity of the extracts, washed platelets were pre-incubated with nitrated or non-nitrated extracts in the presence of 1 mM β-mercaptoethanol (BME) at 37 °C for 6 min to potentially reduce nitration bonds. Platelet aggregation was subsequently induced with TRAP-6 (5 μM) and measured as described above [42].

For assessment of platelet activation, the expression of P-selectin (CD62P) and secretion of CD63 were evaluated by flow cytometry. Washed platelets (200 × 10^9^/L) were pre-incubated for 10 min at 37 °C with nitrated fatty acids from bean extracts (0.5, 1, 2, 4, and 6 mg/mL) or vehicle (0.2% DMSO), followed by stimulation with TRAP-6 (5 μM). A 50 μL aliquot of each sample was then incubated for 30 min at room temperature in the dark with saturating concentrations of anti-CD61-FITC (platelet marker), anti-CD62P-PE (P-selectin), and anti-CD63-PE antibodies. Samples were analyzed using a BD FACSLyric™ flow cytometer (BD Biosciences, San Jose, CA, USA) [47].

### 4.8. Mechanism of Platelet Activation and Secretion Mediated by Mitochondrial Nitrated Compounds

Intracellular calcium levels were measured in washed platelets (5 × 10^7^ platelets/mL) using the calcium sensitive fluorescent dye Fluo-4-AM (0.4 μM). Platelets were incubated with the dye at room temperature for 30 min, followed by a 5 min incubation with either vehicle (0.2% DMSO) or nitrated fatty acids (NO_2_-OA and NO_2_-LN). Fluorescence data were recorded for 15 s to establish a baseline, after which carbonylcyanide p-trifluoromethoxyphenylhydrazone (FCCP, 1 μM) was added to induce calcium mobilization. Data were then collected for an additional 60 s. The effects of the treatments on cytosolic calcium levels were calculated relative to the vehicle control [45,48].

Reactive oxygen species production was assessed in washed platelets (50 × 10^6^ platelets/mL) using 10 μM dihydroethidium (DHE). Platelets were pre-incubated with individual compounds (1 and 2 μM) for 30 min at 37 °C. Antimycin A (20 μM) served as a positive control to increase ROS levels. Fluorescence was analyzed using a BD FACSLyric™ flow cytometer (BD Biosciences, San Jose, CA, USA) [5,49].

Mitochondrial membrane potential (ΔΨm) was evaluated using the cell-permeant dye tetramethylrhodamine methyl ester perchlorate (TMRM, 100 nM). Washed platelets were incubated with 0.2% DMSO (control), NO_2_-fatty acids, or FCCP (1 μM) at 37 °C for 20 min. Changes in ΔΨm were detected by flow cytometry using the BD FACSLyric™ flow cytometer (BD Biosciences, San Jose, CA, USA) [50].

### 4.9. Statistical Analysis

All results are expressed as mean ± standard error of the mean (SEM), based on six independent experiments (n = 6). Statistical analysis was performed using GraphPad Prism 9.0 (GraphPad Software Inc., San Diego, CA, USA). Differences between multiple groups were assessed using one-way ANOVA followed by Bonferroni or Tukey’s post hoc tests, as indicated in the figure legends. Statistical significance was considered at *p* < 0.05, *p* < 0.01, and *p* < 0.001. Comparisons versus the TRAP-6 stimulated control are denoted with # (# *p* < 0.05, ### *p* < 0.001), while comparisons between nitrated and corresponding non-nitrated treatments are indicated with asterisks (*p* < 0.05, * *p* < 0.01, ** *p* < 0.001). Different lowercase letters (a, b, c) denote significant differences among groups according to Tukey’s test (*p* < 0.05), and “ns” indicates no statistically significant difference.

## 5. Conclusions

This work establishes that *Phaseolus vulgaris* L. extracts can be selectively enriched in NO_2_-FAs with potent and mechanistically distinct antiplatelet effects. Nitrated extracts, and particularly NO_2_-LN and NO_2_-OA, consistently suppressed TRAP-6 mediated aggregation, granule secretion, and mitochondrial polarization, effects because of electrophilic nitroalkenes concentrated in the lipid fraction. These findings reveal that even low-lipid food matrices can yield bioactive nitrated species capable of modulating platelet activation pathways without exacerbating cytotoxicity or oxidative stress. While the *in vitro* nature of the work limits direct physiological extrapolation, the data provide a compelling biochemical rationale for considering legumes as precursors of endogenous NO_2_-FAs with cardioprotective properties. Future studies will be required to determine the extent to which such species are formed *in vivo*, their bioavailability, and their contribution to diet-derived modulation of thrombotic risk.

## Figures and Tables

**Table 1:** Fatty acid composition of the *Phaseolus vulgaris* L. (Hallado Alemán variety) bean extract.

Fatty Acid	Per 100 mg
C13:0/Tridecanoic acid	6.66 ±1.30
C14:0/Myristic acid	1.31 ± 0.20
C15:0/Pentadecanoic acid	0.35 ± 0.11
C16:0/Palmitic acid	15.68 ± 1.14
C16:1/Palmitoleic acid	ND
C17:0/Heptadecanoic acid	6.34 ± 1.65
C18:0/Stearic acid	2.18 ± 0.25
C18:1 Δc9 (c18:1 ω9)/Oleic acid	13.76 ± 1.10
C18:1 Δcll/Vaccenic acid	1.24 ± 0.86
C18:2 Δc9, c12 (C18:2 ω6)/Linoleic acid	27.72 ± 0.33
C20:0/Arachidic acid	0.30 ± 0.15
C18:3 Δc9, c12, c15 (C18:3 ω3)/α-Linolenic acid	33.19 ± 0.25
C22:0/Docosanoic acid	0.45 ± 0.12
C23:0/Tricosanoic acid	0.14 ± 0.03
C24:0/Lignoceric acid	0.68 ± 0.05

ND: Not detected.

## Data Availability

The article contains all data necessary to support the conclusion. All data is contained within the article.

## Supplementary Materials

The following supporting information can be downloaded at: https://www.mdpi.com/article/10.3390/molecules31030488/s1, Figure S1: Cytotoxicity by Nitrated vs. Non-nitrated Extracts; Figure S2: Chemical structures of saturated and unsaturated fatty acids identified in the *Phaseolus vulgaris* L. extract; Table S1: Effect of Hallado Alemán Extracts and Their Fractions on Collagen-Induced Platelet Aggregation.

## Abbreviations

The following abbreviations are used in this manuscript:

AA	Antimycin A
ALA	Alpha-linolenic acid
BME	β-mercaptoethanol
CD61	Glycoprotein IIIa (platelet marker)
CD62P	P-selectin
CD63	Lysosomal granule marker
CVD	Cardiovascular disease
DHE	Dihydroethidium
DMSO	Dimethyl sulfoxide
FCCP	Carbonyl cyanide p-(trifluoromethoxy)phenylhydrazone
GC	Gas chromatography
GPCR	G protein-coupled receptor
GPVI	Glycoprotein VI (collagen receptor)
HPLC	High-performance liquid chromatography
IC_50_	Half maximal inhibitory concentration
LA	Linoleic acid
LDH	Lactate dehydrogenase
LN	Linolenic acid
MMP (ΔΨm)	Mitochondrial membrane potential
MUFA	Monounsaturated fatty acid
NO	Nitric oxide
NO_2_-FAs	Nitrated fatty acids
NO_2_-LA	Nitro-linoleic acid
NO_2_-LN	Nitro-linolenic acid
NO_2_-OA	Nitro-oleic acid
OA	Oleic acid
PAR-1	Protease-activated receptor-1
PBS	Phosphate-buffered saline (if needed; appears indirectly)
PPP	Platelet-poor plasma
PRP	Platelet-rich plasma
PUFA	Polyunsaturated fatty acid
ROS	Reactive oxygen species
SEM	Standard error of the mean
SFA	Saturated fatty acid
TMRM	Tetramethylrhodamine methyl ester
TRAP-6	Thrombin receptor-activating peptide 6
UAE	Ultrasound-assisted extraction
