# Supplementary material for: Enhanced Antiplatelet Activity of Nitrated Fatty Acid Extracts from Phaseolus vulgaris L
Source: Molecules. 2026 Jan 30;31(3):488. doi: 10.3390/molecules31030488 (PMC12899701; doi:10.3390/molecules31030488)
Supplement: Supplementary file 1 [file molecules-31-00488-s001.zip › molecules-4069965-supplementary.pdf]

Supporting Information

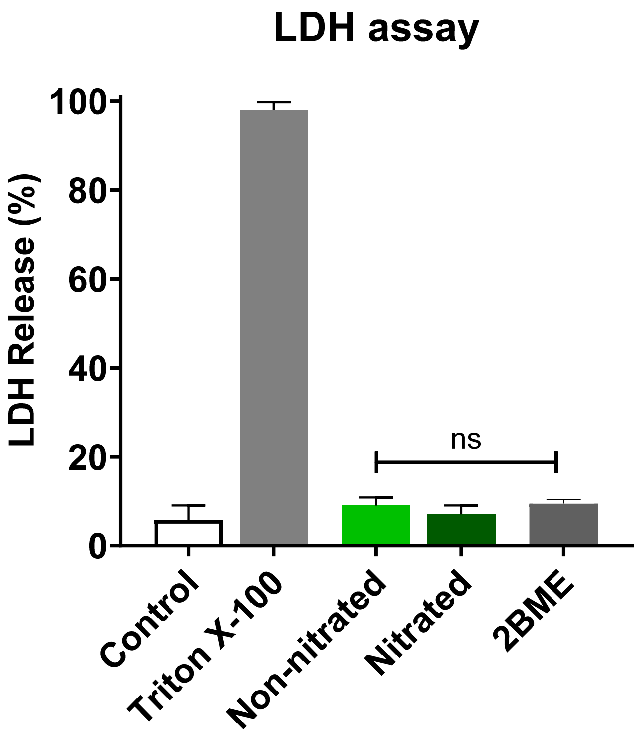

Figure S1 Cytotoxicity by Nitrated vs. Non-nitrated Extracts. LDH release incubated with either nitrated or non-nitrated extract with green as the non-nitrated extract and deep green for the nitrated extract, Triton X-100 as positive control.

Table S1. Effect of Hallado Alemán Extracts and Their Fractions on Collagen-Induced Platelet Aggregation.

	Control (collagen 2 µg/mL)	Nitrated Extract of Hallado	Lipid Fraction of Nitrated Extract of Hallado	Phenolic Fraction of Nitrated Extract	Non-nitrated Extract of Hallado
Aggregation platelets % at 6 mg/mL	65%±4	59±6 ns	61±3 ns	63±5 ns	63±7 ns
Aggregation platelets % at 1 mg/mL	68%±5	62±7 ns	65±6 ns	68±5 ns	65±4 ns

Platelet aggregation in response to collagen (2 µg/mL) with Hallado Alemán extract and fractions. Data are mean ± SD (n = 3–5). No significant differences vs. control (ns, $p > 0.05$).

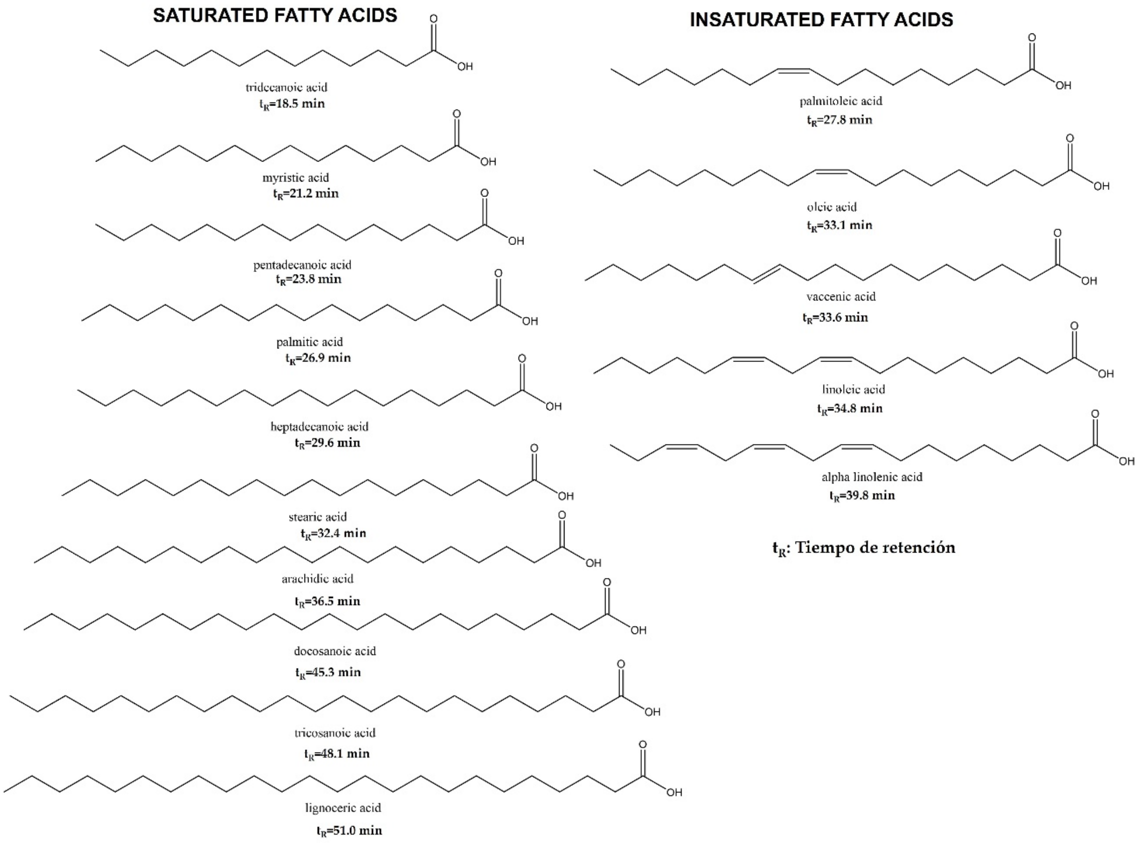

Figure S2 Chemical structures of saturated and unsaturated fatty acids identified in the *Phaseolus vulgaris* L. extract.
